# Validation of Physical Activity Tracking via Android Smartphones Compared to ActiGraph Accelerometer: Laboratory-Based and Free-Living Validation Studies

**DOI:** 10.2196/mhealth.3505

**Published:** 2015-04-15

**Authors:** Eric B Hekler, Matthew P Buman, Lauren Grieco, Mary Rosenberger, Sandra J Winter, William Haskell, Abby C King

**Affiliations:** ^1^Arizona State UniversitySchool of Nutrition and Health PromotionPhoenix, AZUnited States; ^2^Stanford UniversityStanford, CAUnited States

**Keywords:** telemedicine, cell phones, accelerometry, motor activity, validation studies

## Abstract

**Background:**

There is increasing interest in using smartphones as stand-alone physical activity monitors via their built-in accelerometers, but there is presently limited data on the validity of this approach.

**Objective:**

The purpose of this work was to determine the validity and reliability of 3 Android smartphones for measuring physical activity among midlife and older adults.

**Methods:**

A laboratory (study 1) and a free-living (study 2) protocol were conducted. In study 1, individuals engaged in prescribed activities including sedentary (eg, sitting), light (sweeping), moderate (eg, walking 3 mph on a treadmill), and vigorous (eg, jogging 5 mph on a treadmill) activity over a 2-hour period wearing both an ActiGraph and 3 Android smartphones (ie, HTC MyTouch, Google Nexus One, and Motorola Cliq). In the free-living study, individuals engaged in usual daily activities over 7 days while wearing an Android smartphone (Google Nexus One) and an ActiGraph.

**Results:**

Study 1 included 15 participants (age: mean 55.5, SD 6.6 years; women: 56%, 8/15). Correlations between the ActiGraph and the 3 phones were strong to very strong (ρ=.77-.82). Further, after excluding bicycling and standing, cut-point derived classifications of activities yielded a high percentage of activities classified correctly according to intensity level (eg, 78%-91% by phone) that were similar to the ActiGraph’s percent correctly classified (ie, 91%). Study 2 included 23 participants (age: mean 57.0, SD 6.4 years; women: 74%, 17/23). Within the free-living context, results suggested a moderate correlation (ie, ρ=.59, *P*<.001) between the raw ActiGraph counts/minute and the phone’s raw counts/minute and a strong correlation on minutes of moderate-to-vigorous physical activity (MVPA; ie, ρ=.67, *P*<.001). Results from Bland-Altman plots suggested close mean absolute estimates of sedentary (mean difference=–26 min/day of sedentary behavior) and MVPA (mean difference=–1.3 min/day of MVPA) although there was large variation.

**Conclusions:**

Overall, results suggest that an Android smartphone can provide comparable estimates of physical activity to an ActiGraph in both a laboratory-based and free-living context for estimating sedentary and MVPA and that different Android smartphones may reliably confer similar estimates.

## Introduction

Reduced sitting time and increased moderate-to-vigorous physical activity (MVPA) confers an array of health benefits [1-3]. Identifying cost-efficient solutions for tracking physical activity passively has become an important scientific objective [4]. Previous research on activity monitoring has focused on devices dedicated solely to this purpose, such as the ActiGraph accelerometer (ActiGraph, Fort Walton, FL, USA). Indeed, this trend of dedicated activity monitoring devices has continued with consumer devices such as the Fitbit, Jawbone UP, and Misfit Shine, among others. There is also increasing interest in improving physical activity detection through multiple sensors [5,6], but these added sensors (eg, heart rate, global positioning systems) often impact the ability to enable long-term monitoring [4] in context.

One potentially cost-efficient and low-burden mechanism for tracking daily physical activity is the smartphone [6,7]. The smartphone includes a variety of advantages that make it an excellent stand-alone device. Specifically, smartphones include a variety of sensors, such as built-in accelerometry and global positioning system (GPS), increasingly powerful computing capabilities, large data capacity, wireless connectivity to other sensors (eg, connectivity to weight scales or blood pressure monitors), and Internet access. These technical capabilities allow smartphones to track, process, and send physical activity information while also functioning as a “hub” for other health information [7,8]. Beyond these technical capabilities, smartphones often accompany individuals throughout the day and, thus, easily fit into an individual’s daily routine.

At present, there is relatively little systematic research exploring how accurately physical activity can be tracked via smartphones. Some studies have explored the utility of ecological momentary assessment (EMA) via smartphones [9], activity recognition using machine learning/neural network analyses [10-13], and activity classification via an iPod Touch for older adults [14]. There remain many unresolved questions related to tracking physical activity via smartphones. For example, although there was a study examining tracking among older adults, the vast majority of research is conducted among younger cohorts. The ActiGraph is a well-validated accelerometer commonly used in epidemiological, surveillance, and intervention research to quantify physical activity [1,15-18]. As such, a comparison of physical activity estimates collected via smartphone versus ActiGraph accelerometry would provide insights into whether a common smartphone could provide estimates of physical activity with similar levels of accuracy to this common field-based assessment strategy. This would be valuable based on the large body of research linking these cut-point-based estimates of sedentary, light, and moderate-to-vigorous levels of physical activity to health outcomes [1,15-18]. These data are largely absent for more advanced machine learning techniques of activity classification; thus, comparison to cut-point-based estimates is a scientifically important question until the health linkages to the more advanced analytic techniques can be made.

The purpose of this work was to determine the validity and reliability of Android smartphones for tracking physical activity utilizing cut-point–based methods of activity classification. In particular, we sought to determine the validity of Android smartphones for categorizing physical activity into sedentary, light physical activity, and moderate-to-vigorous levels of physical activity in a laboratory setting. We also explored interdevice reliability by comparing the estimates from 3 Android phones used simultaneously in the laboratory. Finally, we sought to determine the validity of the smartphones by comparing daily estimates of physical activity between the ActiGraph and an Android smartphone in a free-living context.

## Methods

### Overview

We conducted both a laboratory-based and free-living study. In the laboratory-based study (study 1), individuals engaged in prescribed activities of various intensities over a 2-hour period wearing both an ActiGraph and 3 Android smartphones (ie, HTC MyTouch, Google [manufactured by HTC] Nexus One, and Motorola Cliq) all worn both on the hip and in the pocket. In the free-living study (study 2), individuals engaged in usual daily activities that were tracked over a 7-day period via an Android smartphone (Google Nexus One) and an ActiGraph.

### Study 1: Laboratory Study

#### Participants

Participants were a convenience sample of 15 midlife and older adults living in the San Francisco Bay area in California. Midlife and older adults (ie, aged 40 or older) were chosen because they represent an understudied population for activity classification and because the algorithms developed were explicitly being developed for a smartphone-based physical activity intervention [8]. Participants were recruited via word of mouth, advertisements, and email listservs. Participants completed the Physical Activity Readiness Questionnaire (PAR-Q) to ensure that they could safely engage in physical activity [19].

#### Procedures

The procedures for this study were similar to previously published work conducted by the study investigators [20]. Specifically, all participants completed a written informed consent that was approved by Stanford University’s Human Use Committee. Participants were asked to wear an ActiGraph GT3X+ accelerometer on their nondominant hip along with the 3 Android smartphone phones also on the nondominant hip. Participants were asked to wear clothing to the laboratory session that was suitable for exercise and had pockets.

The ActiGraph GT3X+ is a small, electronic, triaxial device that is worn on the waist and measures activity “counts” (epoch set to raw for the 3 axes for this study but converted to 1-minute values with just the vertical axis for comparison with previously validated cut-point calculations) [1,21,22]. The 3 smartphones were chosen because at the time (2009) they were common Android devices and because comparison between the 3 phones would provide insights about reliability both within and between manufacturers. The Android platform was chosen because it allowed the built-in accelerometer to run continuously and because there is increased use of the Android phones, particularly among lower income groups.

**Figure 1:**
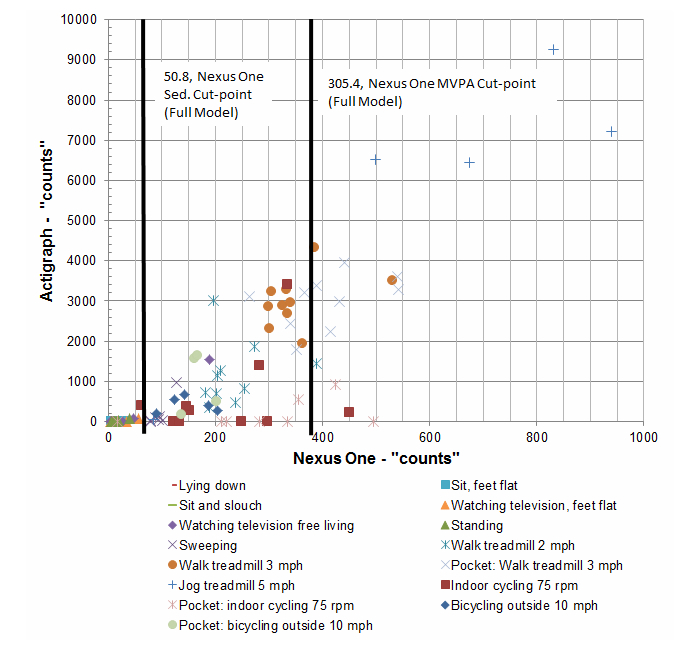
Phone to ActiGraph comparison across activities (laboratory study).

We chose to include 3 different Android smartphones to gauge the reliability and validity across manufacturers (ie, Motorola vs HTC) and within manufacturer by using different firmware versions of Android (ie, Nexus One vs MyTouch). After attaching the devices, participants were asked to engage in a series of activities while wearing the ActiGraph and the Android phones (see Figure 1 for a list of activities). Each activity was conducted for 5 minutes followed by a transition period to the next activity. The overall protocol lasted approximately 2 hours per participant. For some activities, particularly running on the treadmill at 5 mph, participants were given the option to opt out if they were not able to accomplish it safely.

A conversion was required to translate the raw values gathered from the smartphones into values similar to ActiGraph “counts.” To calculate these phone-based counts, the first step was the use of a low-pass filter to account for the effects of gravity, followed by the calculation of an area under the curve measurement that represented total movement detected by the accelerometers. This technique is commensurate with previously published work [20].

#### Data Processing

Standard data processing techniques for calculating cut-point-derived estimates of sedentary, light physical activity, and moderate/vigorous levels of physical activity were used for processing the ActiGraph data based on previously published work on meaningful cut-points among older adults for sedentary (<100 counts) and moderate-to-vigorous intensity physical activity (>1951) [1]. These included setting the sampling rate for the ActiGraph to 80 Hz and gathering raw data across the 3 axes, which was then converted into counts using ActiLife 6.1 software (ActiGraph, Pensacola, FL, USA) based on the vertical axis. For the phone data, a custom app was developed that allowed for raw acceleration values similar to ActiGraph counts to be attained. For all phones, the custom app sampled at the maximum rate allowed for each phone (ie, 20 Hz for the HTC MyTouch and Google Nexus One; 80 Hz for the Motorola Cliq).

#### Statistical Analyses

We first calculated an intraclass coefficient (ICC) reflecting the effect of individual variability on observed counts. Our analyses found that the within-subject variance was effectively zero across all possible comparisons using a leave-one-out cross-validation method for each mixed model analysis described subsequently. Based on this and that the counts were not normally distributed, Spearman rank order correlations were deemed an acceptable method for determining these associations. Mixed model analyses [23] were conducted to create a regression equation comparing each phone to the ActiGraph. The regression equations were validated utilizing a leave-one-out cross-validation procedure for each subject’s activity as has been used in previous research [21]. Because the cut-point-based classification via the ActiGraph is known to have difficulties with properly estimating cycling activities and standing [24], and because phones worn in the pocket would likely affect the phone’s counts, a variety of datasets with different filtering criterion were explored (eg, hip-only data, no biking and standing activities included).

As previously mentioned, we used fairly standardized cut-point values for ActiGraph counts of sedentary physical activity (ie, ActiGraph counts <100) [1,25] and MVPA (ie, ActiGraph counts >1952 [26]) because they have been validated among older adults specifically [1]. We utilized our regression equation (ie, phone cut-point values= beta*[imputed ActiGraph cut-point values] + intercept) to convert the validated ActiGraph cut-points into Android phone cut-points for the phone counts. We then utilized these derived phone-based cut-points to then label each minute of measured data via the phones as sedentary, light physical activity, or moderate-to-vigorous levels of physical activity.

We used standard conventions for labeling the strength of our Spearman correlations (ie, very weak=0-.19, weak=.2-.39, moderate=.4-.59, strong=.6-.79, and very strong=.8-1.0 [27]). Within the laboratory study, we had known activities with known metabolic equivalent (MET) values based on the Compendium of Physical Activities [28]. We calculated the percentage of total observations each phone correctly classified each activity as sedentary, light, or moderate-to-vigorous. To support interpretation of these percentages, we utilized the percent correctly classified by the ActiGraph minus 5% as the range for acceptable percent classification, similar to previous work [15]. For example, if the ActiGraph correctly classified 70% of total activities, the comparable range for the phones would be 65% or greater.

### Study 2: Free-Living Study

#### Participants

Participants were a subsample from the previously reported Mobile Interventions for Lifestyle Exercise and eating at Stanford (MILES) study and, thus, recruitment procedures have been reported previously [8]. In brief, the target population consisted of community-dwelling adults in the San Francisco Bay area aged 45 years and older who were insufficiently physically active (ie, engaged in less than 60 minutes of self-reported MVPA per week), self-reported typically sitting for 10 or more hours per day, were able to participate safely in a physical activity program based on the PAR-Q [19], and were currently using a mobile phone but not a smartphone. In addition, participants were excluded if they did not provide sufficient data to complete the analyses (eg, insufficient wear time of the phone or ActiGraph; see data processing described subsequently).

#### Procedures

For this validation study, only the baseline phase (ie, the first week when no intervention was provided) from the MILES intervention study was used. Participants were requested to continue with their normal physical activity during the baseline phase. All participants (N=23) were provided a Nexus One smartphone equipped with a custom app for tracking physical activity via the built-in accelerometer and also wore an ActiGraph (GT3X+), utilizing standard quality control procedures for the ActiGraph [29]. Participants wore the ActiGraph accelerometer for at least 7 days (our request was 7 days, but due to scheduling issues some individuals wore the ActiGraph longer). Participants were asked to wear the ActiGraph and smartphone at the same times during waking hours. Participants wore the ActiGraph on their hip and were allowed to wear the smartphone either on the hip or in their pocket. After the baseline week, participants reported where they wore the phone and exploratory analyses suggested that placement did not influence any of the results (which were in-line with our laboratory-based study as described subsequently).

#### Data Processing

Data compliance and cleaning procedures for the ActiGraph and smartphone were consistent with other large-scale cross-sectional accelerometer studies [25,30], such that (1) valid hours of data consisted of no more than 60 consecutive “zero” values (interpreted as nonwear time) and (2) a valid day was defined as at least 10 valid hours/day for both the Nexus One phone and the ActiGraph. We included this 10-hour stipulation for both phones to ensure comparable wear time (note: in separate analyses not reported, there were no significant differences in wear time between the ActiGraph and Nexus One phone and individuals did wear them at the same times throughout the day). We did not include a stipulation on the number of valid days an individual must complete to be part of the study. Instead, we included any day that included both valid ActiGraph and smartphone accelerometry data. The same cut-points that were used in the laboratory-based study were used in the free-living study for the ActiGraph [26,31] and the phone cut-points were based on the regression equations calculated in the laboratory-based study.

#### Statistical Analyses

Although ICC were originally used, we again found minimal within-person variation. As such, Spearman rank order correlations were calculated between the ActiGraph and smartphone. In addition, Bland-Altman plots [32] were created to compare the difference between the estimates of minutes in each activity intensity per day between the ActiGraph and the smartphone utilizing similar procedures reported previously [29]. As is convention with Bland-Altman plots, a criterion level of agreement that would be expected is required for proper interpretation of the mean difference and variance calculation. We assumed that a mean difference of less than 10 minutes constituted agreement for MVPA based on national guideline recommendations suggesting clinically meaningful activity occurring past the 10-minute threshold [3]. There is no consensus on what “meaningful” differences in sedentary or light activity might be. Nonetheless, based on our own previous work [1], we postulated that mean differences greater than 60 minutes for sedentary and light activity would suggest poor agreement. For this study, we did not have a “gold-standard” measure of sedentary, light physical activity, and moderate-to-vigorous levels of physical activity but instead a well-validated field measure, the ActiGraph. Without a gold-standard metric, it is hard to determine what the “right” answer should be. This is important to consider with regard to interpretation of the confidence intervals because wide confidence intervals could occur based on error from both the ActiGraph and the smartphone. As such, although we report confidence intervals in our Bland-Altman plots, we suggest caution in overinterpretation of these confidence intervals.

## Results

### Study 1: Laboratory Study

There were a total of 15 participants (age: mean 55.5 years, SD 6.6, range 43-65; women: 56%, 8/15; education: mean 16.25 years, SD 1.6) who participated in study 1. A list of the activities in the laboratory study can be found in Figure 1. Table 1 reports Spearman rank order correlations between the ActiGraph counts and the 3 phones. Overall, results suggest strong correlations between the ActiGraph and the 3 phones and between the 3 phones (ρ*=*.90). Figure 1 is a visual representation of all activities with color-coded labeling to identify the classification of each activity as sedentary, light physical activity, or MVPA.

**Table 1:** Spearman rank order correlations^a^ (ρ) between raw ActiGraph and raw phone counts for the laboratory study (N=15).

Statistical model by activity monitoring device^b^		Cliq		MyTouch		Nexus One	
		ρ	*P*	ρ	*P*	ρ	*P*
**Full model**							
	ActiGraph	.77	<.001	.82	<.001	.80	<.001
	Cliq			.95	<.001	.90	<.001
	MyTouch					.90	<.001
**No bike & standing**							
	ActiGraph	.85	<.001	.89	<.001	.83	<.001
	Cliq			.93	<.001	.88	<.001
	MyTouch					.87	<.001
**Hip only**							
	ActiGraph	.82	<.001	.86	<.001	.85	<.001
	Cliq			.93	<.001	.95	<.001
	MyTouch					.93	<.001
**Hip only and no bike & standing**							
	ActiGraph	.81	<.001	.85	<.001	.83	<.001
	Cliq			.92	<.001	.92	<.001
	MyTouch					.89	<.001

^a^ The Spearman correlations are between counts/min derived for the ActiGraph and the 3 Android smartphones (Motorola Cliq, HTC MyTouch, and Google/HTC Nexus One).

^b^ The different models correspond to different filters (ie, no bike & standing excludes bicycling and standing; hip-only excludes measures whereby the phones were in the pocket).

**Figure 2:**
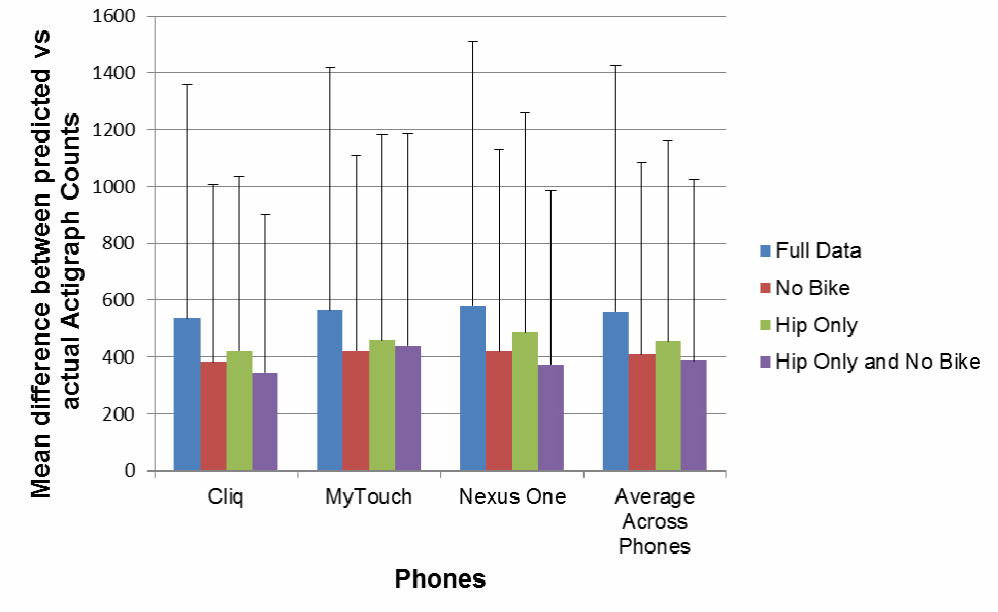
Comparison of the stability of different regression model estimates (laboratory study). The error bars represent the root mean standard error across all models run utilizing a leave one out procedure.

Table 2 reports results of the mixed model regression equations for each cut-point estimate. The betas and intercepts were the values used to calculate the phone-based cut-points listed when the ActiGraph cut-points (ie, <100 and >1952) were imputed into the equation. Figure 2 provides estimates of the mean differences between the predicted values from the phones and the actual ActiGraph counts across phones and across different modeling datasets (eg, the full dataset, to the dataset that only included hip data and excluded biking/standing). Overall, there was some instability in the overall estimate depending on the observations included/excluded from the models using the leave-one-out technique based on the large root mean standard errors but that these differences were not greatly impacted by the specific phone used or the filtering strategies. Overall, the models were improved if data were aggregated across all phones as opposed to using phone-specific estimates. For the remainder of the paper, we only refer to the aggregated estimates of cut-points across phones as these appeared most stable and we continue to report both the “full model” and “no bike and standing” models because there appeared to be improved model fit using this filter but no improved model fit when excluding measurements from the phones while worn in the pocket.

**Table 2:** Regression equations and cut-points for sedentary and moderate-to-vigorous levels of physical activity in the laboratory.

Phone		Intercept^a^	b^a^	N	Sedentary cut-point^b^	MVPA cut-point^c^
**Full data**						
	Moto Cliq	–260.34	6.73	169	53.54	328.72
	HTC MyTouch	–304.62	7.96	169	50.82	283.46
	Google Nexus One	–247.00	7.15	169	48.56	307.76
	All phones mean	–270.65	7.28		50.92	305.36
**No bike & standing**						
	Moto Cliq	–182.81	7.63	133	37.06	279.77
	HTC MyTouch	–205.17	8.67	133	35.20	248.86
	Goo/HTC Nexus One	–171.66	8.14	133	33.36	260.75
	All phones mean	–186.55	8.15		35.17	262.47

^a^ The betas and intercepts were developed as an aggregation of the results of the leave-one-out technique (ie, averaging the beta and intercept estimates from all models).

^b^ The cut-point value imputed into the regression equation for the sedentary cut-point was <100.

^c^ The cut-point value imputed into the regression equation for the MVPA cut-point was >1951.

Figure 2 shows the mean difference between the predicted value of an ActiGraph count compared to the actual ActiGraph count (ie, predicted count – actual count). This mean difference across different models allows for an estimate of differences across models when using the leave-one-out strategy of model building. Higher mean differences suggest less stable regression models that are more influenced by individual observations in the model. The error bars represent the root mean standard error across all models run utilizing a leave-one-out procedure, which further provides insights on the interpretability of the models. High root mean standard errors suggest large variation in estimates during the leave-one-out procedure, but also provide an estimate of “meaningful” mean difference estimates across the various regression models. Results suggest that the mean differences observed did not differ by phone or data-filtering strategy used, but that there was large variation of impact across observations, thus justifying the use of the leave-one-out method for creating a more stable regression model.

Table 3 reports the percentage of time each device accurately classified an activity into its corresponding MET classification (ie, sedentary, light physical activity, or MVPA) [28]. Overall, results suggest that the phones correctly classified activities at nearly the same level as the ActiGraph. For example, when excluding behaviors from the model that are known to be poorly classified using cut-point estimates (ie, bicycling and standing), the ActiGraph correctly classified 91% (108/119) of all activities across all participants. In comparison, the phones correct classification levels ranged from 78% (73/93; MyTouch, Full Model) to 91% (85/93; Nexus One, no bike and standing model). Not surprisingly, cycling activities and standing exhibited the poorest estimated agreement (see Table 3). Interestingly, the phones actually did better at correctly classifying the light intensity activity of sweeping. Again, Figure 1 provides a visual summary of these classifications on a labeled scatterplot for the Nexus One.

**Table 3:** Correct classification of activity intensity level for each device.

Activity	Placement	Correct classification,^a^ %						
		Nexus One		MyTouch		Cliq		ActiGraph
		Full	No bike & stand^b^	Full	No bike & stand^b^	Full	No bike & stand^b^	N/A
Overall		69%	73%	59%	60%	63%	65%	64%
Overall excluding behaviors^b^		90%	91%	78%	80%	83%	83%	91%
Bicycling outside 10 mph	Hip	0%	0%	0%	0%	0%	0%	0%
Bicycling outside 10 mph	Pocket	0%	0%	0%	0%	0%	0%	0%
Cycling indoors 75 rpm	Hip	18%	36%	0%	0%	0%	9%	7%
Cycling indoors 75 rpm	Pocket	50%	63%	63%	63%	63%	88%	0%
Lying down	Hip	91%	91%	82%	82%	82%	82%	100%
Sitting while slouching	Hip	100%	100%	89%	89%	89%	89%	100%
Sitting with back straight	Hip	100%	100%	80%	80%	90%	90%	100%
Television (free-movement)	Hip	91%	82%	82%	73%	82%	73%	93%
Television (sitting straight)	Hip	90%	90%	80%	80%	80%	80%	100%
Standing Straight	Hip	9%	18%	0%	0%	9%	9%	0%
Sweeping	Hip	100%	100%	100%	100%	100%	100%	50%
Treadmill 2 mph	Hip	90%	80%	90%	90%	90%	90%	92%
Treadmill 3 mph	Hip	80%	100%	20%	40%	80%	90%	92%
Treadmill 3 mph	Pocket	80%	90%	90%	90%	70%	70%	91%
Treadmill 5 mph	Hip	80%	80%	80%	80%	60%	60%	80%

^a^ These values are the percentage of times the activity was correctly categorized according to its physical activity intensity level (ie, sedentary, light, or moderate-to-vigorous intensity physical activity) by each of the 4 devices. For this work, we explored correct classification both using different phones and via different cut-point algorithms based on different regression models. The cut-points used here were the average cut-point estimates across all the phones for both the full model cut-points (ie, <50.92 for sedentary and >305.36 for MVPA) and the model generated when biking and standing was excluded when creating the cut-point estimates (ie, “no bike & standing” model cut-points were <35.17 for sedentary and >262.47 for MVPA). For the full model, N=132.

^b^ This overall estimate of correct classification excluded the following behaviors that are known to be problematic for classifying using a cut-point strategy: bicycling outdoors, indoor cycling, and standing still (N=93).

### Study 2: Free-Living Study

There were 23 participants who had acceptable data for comparing the phone accelerometer to the ActiGraph (age: mean 57.0 years, SD 6.4, range 45-69; women: 74%, 17/23; body mass index: mean 29.5, SD 5.9, range: 20.8-40.9; white: 70%, 16/23; Asian/Asian American: 22%, 5/23; bachelor’s degree or higher: 79%, 18/23; married: 44%, 10/23; working full time: 64%, 14/23). On average, participants had approximately 5 days of valid wear time for both the ActiGraph and smartphone accelerometry data (days per participant: mean 4.8, SD 2.1; total days=107*)*. Table 4 reports correlations between the estimated number of minutes engaged in sedentary, light physical activity, and moderate-to-vigorous levels of physical activity based on the Nexus One phone using the full model cut-points derived from study 1 compared to the ActiGraph (listed in Figure 1). Overall, results suggested moderate to strong correlations between the direct estimates for sedentary physical activity and MVPA (eg, raw count comparisons: ρ=.44, *P*<.001; sedentary: ρ=44, *P*<.001*;* MVPA: ρ=.67, *P*<.001) and weak correlations for light physical activity (ρ=.38, *P*<.001). These correlations were nearly the same as the no bike and standing correlations (eg, raw count comparisons: ρ*=.35, P*<.001; sedentary: ρ*=*.44, *P*<.001; light: ρ=.34, *P*<.001; MVPA: ρ=.68, *P*<.001).

**Table 4:** Free-living Spearman rank correlations between ActiGraph and NexusOne smartphone.

Actigraph	Smartphone^a^							
	Raw count		Sedentary		Light		MVPA	
	ρ	*P*	ρ	*P*	ρ	*P*	ρ	*P*
Raw count	.59	<.001	–.22	.02	.32	<.001	.57	<.001
Sedentary	–.34	<.001	.44	<.001	.11	.27	.00	.98
Light	.14	.16	–.13	.20	.38	<.001	–.07	.49
MVPA	.54	<.001	–.21	.03	.06	.53	.67	<.001

^a^ Smartphone estimates of min/day in each category are based on the “full” model average cut-points that were derived from study 1.

**Figure 3:**
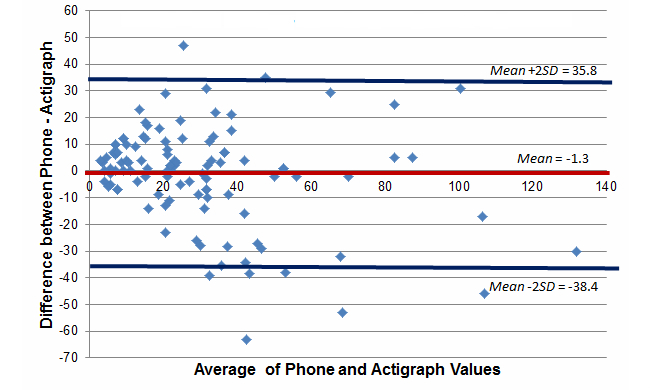
Bland-Altman plot comparing estimated minutes of MVPA per day between the phone and ActiGraph, full model (free-living study).

**Figure 4:**
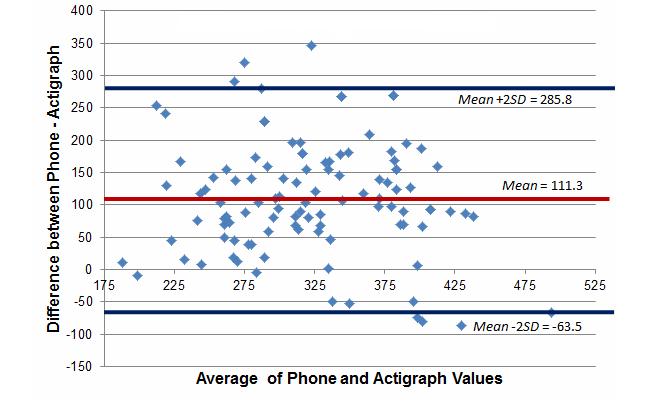
Bland-Altman plot comparing estimated minutes of light activity per day between the phone and ActiGraph, full model (free-living study).

**Figure 5:**
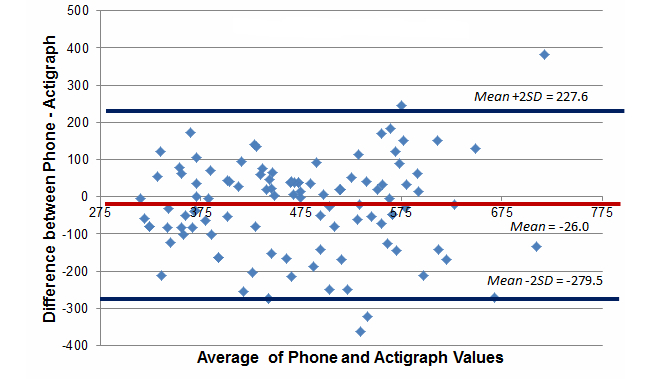
Bland-Altman plot comparing estimated minutes of sedentary behavior per day between the phone and ActiGraph, full model (free-living study).

Figures 3-5 report Bland-Altman plots comparing the ActiGraph and smartphones utilizing the full model cut-point estimates. Comparison of the plots suggest good absolute mean-level differences in sedentary and MVPA activity minutes/day estimates (sedentary: mean difference=–26.0 min/day, 95% CI –279.5 to 227.6; MVPA: mean difference=–1.3 min/day, 95% CI –38.4 to 35.8). Absolute mean-level differences for light physical activity were outside of the acceptable range (mean difference=–111.2 min/day, 95% CI –285.8 to –63.5). Although not shown, we did create Bland-Altman plots for the no bike and standing models and consistently found poorer absolute estimates suggesting that for absolute estimates in a free-living context, the full model cut-points listed in Table 2 (ie, <50.92 for sedentary and >305.36 for MVPA) were superior. Based on the lack of a gold standard in this study, the 95% confidence intervals are not as easily interpreted because the error likely comes from measurement issues with both devices. As such, they are provided more for broader context but should be interpreted with caution.

## Discussion

### Principal Findings

The purpose of this work was to determine the validity and reliability across different Android smartphones for tracking physical activity among midlife and older adults. Overall, results indicated (1) Android smartphone raw counts are strongly correlated to ActiGraph counts in a laboratory-based and free-living setting suggesting the accelerometers provide similar estimates regardless of the activity classification algorithm used, (2) Android smartphone raw counts were strongly correlated with one another suggesting that different Android phones reliably provide similar estimates before any algorithm, (3) placement in the pocket versus on the hip both in a laboratory-based and free-living context did not detrimentally impact estimates suggesting that phones can reliably be worn in either location, and (4) absolute classifications of activity intensity between the phone and ActiGraph were comparable both in-laboratory (based on similar correct classification percentages of known activities) and free-living context (based on absolute mean difference estimates via Bland-Altman plots) for sedentary and MVPA estimates but not light physical activity.

Results indicating that the phone accelerometers and ActiGraph are strongly correlated have important implications for not only cut-point-based activity classification strategies, but also any classification strategy utilizing an accelerometer. In particular, because the effective raw signals are providing similar information, it is quite plausible that similar algorithm strategies can be used between devices with similar effects, assuming some degree of calibration. This is important because it suggests other activity recognition strategies, such as machine learning/neural network analyses [10-13], can likely provide similar estimates from the raw accelerometer signals across phones. This also increases confidence that estimates between Android phones from apps that utilize the accelerometer for activity classification will likely be comparable across phones.

Results suggesting phone placement did not impact sedentary and MVPA estimates have important implications for usability. Specifically, we found an almost even split in preferences related to wearing the phone, with some desiring to wear it on the hip and others desiring to keep it in their pockets. A phone will likely be worn if it fits into a person’s daily life. As such, allowing individuals to wear the device in whatever way they so desire increases the likelihood of continuing to gather the data in the long-term (as we did in our MILES intervention trial [8]). Our results suggest that acceptable activity estimates can be acquired either on the hip or in the pocket. Further work should continue to explore ways to further improve “wearability” of a system. This interest is evident in the commercial sector with devices such as the Fitbit Flex, Jawbone UP, or Misfit Shine, which are all wrist-worn activity monitors.

Finally, our results suggesting that the phones gave similar absolute estimates of sedentary and MVPA activities to an ActiGraph in both in-laboratory and free-living studies has important implications for epidemiologic and intervention research. At present, there is far more data supporting the linkage between cut-point-derived estimates of physical activity with health outcomes compared to the other data analytic techniques [1,15,33]. Based on this, although it is likely the machine learning-based classification techniques will eventually provide better methods for classifying physical activity, at present, there is still value in focusing on validating the smartphone compared to the classical cut-point-derived estimates from the ActiGraph. Our results suggest that the phones can provide an acceptable alternative to an ActiGraph for classifying sedentary and MVPA, but not light physical activity.

### Limitations

The ActiGraph was utilized as our primary comparison metric. Although the ActiGraph is acceptable for field-based research, it is not a gold-standard activity monitoring device. This is particularly important to be mindful of when interpreting the Bland-Altman plots because the Bland-Altman plots are designed to support comparison of a new measure to a previous gold-standard metric. The Bland-Altman plots revealed good mean-level differences but poor confidence interval estimates between the smartphone and ActiGraph. Because the ActiGraph is not a gold-standard measure, it is quite plausible that the high confidence intervals can be attributed not only to variance from the smartphone but also the ActiGraph. As such, the confidence intervals from the Bland-Altman plots need to be interpreted with caution. Further, the ActiGraph cut-points utilized incorporated only the vertical axis whereas the phones used all 3 axes, thus establishing another potential source of error between the two.

A second limitation of our work is that we utilized a cut-point-based strategy for activity classification rather than newer strategies such as neural network analysis or machine learning techniques [10-13]. Although a cut-point strategy does not afford the level of precision that these newer techniques do, cut-point estimates are still pragmatic to use in both epidemiologic and intervention research. In particular, the national guidelines for physical activity do not focus on specific behaviors, but instead the broad classes of moderate and vigorous intensity physical activity that we can classify with cut-point algorithms [3]. As such, intensity classification has utility for physical activity recommendations because it more directly corresponds with national guidelines. In addition, there is also much more work linking cut-point-based estimates of physical activity to health outcomes compared with the more precise activity classification techniques [1,33]. As such, the regression equations generated provide a strategy for establishing a linkage from estimates derived from smartphones to those health outcome linkages made via previous work that found associations with ActiGraph estimates via cut-point algorithms and health outcomes.

Another limitation is the brands of the smartphones we studied. In 2009, HTC and Motorola were among the most important manufacturers of Android smartphones, but at this point Samsung has become the primary Android phone manufacturer. Unfortunately, our study cannot provide any insights on the quality of Samsung devices. Further, although the data were comparable across our phones, it is impossible to determine if the phone estimates would remain comparable in newer phones. That said, based on the plethora of different Android phones available, our data at least increases confidence that different manufacturers and running different firmware versions of Android may provide similar estimates of physical activity based on the accelerometry.

Strengths of this research were that data are available from both an in-laboratory and free-living context. Further, the samples used to create the estimates are in-line with other physical activity assessment/validation protocols. Finally, validation occurred via direct observation of activities in laboratory.

### Conclusions

Overall, our results suggest that an Android smartphone appears to be an acceptable alternative for estimating sedentary and moderate-to-vigorous intensity physical activity to an ActiGraph accelerometer in both a laboratory-based and free-living context. This suggests that smartphones might be an effective mechanism, by themselves, for tracking physical activity. Future work should explore other potential analytic techniques (eg, machine learning) for further improving the classification.

## Abbreviations

EMA: ecological momentary assessment
GPS: global positioning system
MET: metabolic equivalent
MILES: Mobile Interventions for Lifestyle Exercise and eating at Stanford
PAR-Q: Physical Activity Readiness Questionnaire
MVPA: Moderate-to-Vigorous Physical Activity
ICC: intraclass coefficient
